# Towards Safer Medication Use in Older Adults: Investigating Barriers and Facilitators to Deprescribing

**DOI:** 10.1093/geroni/igaf122.3388

**Published:** 2025-12-31

**Authors:** Madhavi Eerike, Gomathi Ramaswamy, Priyadharsini Rajendran, Paul Mathai, Veena Nayak, Vijay Kumar Karra

**Affiliations:** All India Institute of Bibinagar Hyderabad, Hyderabad, Telangana, India; All India Institute of Bibinagar Hyderabad, All India Institute of Bibinagar Hyderabad, Telangana, India; Jawaharlal Institute of Postgraduate Medical Education and Research, Pondicherry, Puducherry, India; Malabar Medical College Hospital & Research Centre, Modakkallur, Kerala, India; Kasturba Medical College, Manipal, Manipal, Karnataka, India; All India Institute of Bibinagar Hyderabad, All India Institute of Bibinagar Hyderabad, Telangana, India

## Abstract

**Background:**

Deprescribing, the systematic reduction or discontinuation of unnecessary medications, is crucial for optimizing pharmacotherapy in older adults. However, its implementation faces challenges, including systemic barriers, knowledge gaps, and patient-specific complexities. Understanding healthcare professionals’ perspectives is vital to improving deprescribing practices and enhancing elderly patients’ health outcomes.

**Methods:**

This qualitative study employed in-depth interviews with healthcare professionals (n = 52) to explore barriers, facilitators, and potential improvements in deprescribing. Thematic analysis was used to identify key insights and patterns from the collected data.

**Results:**

Key barriers included limited access to healthcare services, particularly in rural and underserved areas, and knowledge gaps in geriatric pharmacology among resident doctors. Patient-specific challenges, such as advanced age and multiple comorbidities, compounded these difficulties. Chronic use of medications like proton pump inhibitors (PPIs) and benzodiazepines was a prominent obstacle. Systemic issues, such as inefficient workflows and poor interdisciplinary coordination, also hindered deprescribing. Facilitators included patient acceptance and active participation in shared decision-making, supported by effective communication skills. The integration of electronic health records (EHRs) enhanced continuity of care and medication tracking. Increasing acceptance of deprescribing among doctors, especially in government healthcare settings, was also observed.

**Conclusion:**

Addressing systemic barriers, enhancing patient education, and implementing structured deprescribing guidelines are essential for improving practices. Strengthening interdisciplinary collaboration and leveraging digital tools, such as EHRs, can support safe and effective medication discontinuation. These findings underscore the need for targeted interventions to optimize deprescribing and improve health outcomes for elderly patients.

